# Case Report: Neutralization of Autoantibodies Targeting G-Protein-Coupled Receptors Improves Capillary Impairment and Fatigue Symptoms After COVID-19 Infection

**DOI:** 10.3389/fmed.2021.754667

**Published:** 2021-11-18

**Authors:** Bettina Hohberger, Thomas Harrer, Christian Mardin, Friedrich Kruse, Jakob Hoffmanns, Lennart Rogge, Felix Heltmann, Michael Moritz, Charlotte Szewczykowski, Julia Schottenhamml, Martin Kräter, Antonio Bergua, Matthias Zenkel, Andreas Gießl, Ursula Schlötzer-Schrehardt, Robert Lämmer, Martin Herrmann, Annekathrin Haberland, Peter Göttel, Johannes Müller, Gerd Wallukat

**Affiliations:** ^1^Department of Ophthalmology, Universitätsklinikum Erlangen, Friedrich-Alexander-University Erlangen-Nürnberg, Erlangen, Germany; ^2^Max-Planck-Zentrum für Physik und Medizin, Max Planck Institute for the Science of Light, Erlangen, Germany; ^3^Department of Internal Medicine 3, Universitätsklinikum Erlangen, Friedrich-Alexander-University Erlangen-Nürnberg, Erlangen, Germany; ^4^Berlin Cures GmbH, Berlin, Germany

**Keywords:** functional GPCR autoantibodies, COVID-19, long-COVID syndrome, chronic fatigue syndrome, BC 007, OCT angiography, glaucoma

## Abstract

Clinical features of Coronavirus disease 2019 (COVID-19) are caused by severe acute respiratory syndrome coronavirus-2 (SARS-CoV-2). Acute infection management is a substantial healthcare issue, and the development of long-Covid syndrome (LCS) is extremely challenging for patients and physicians. It is associated with a variety of characteristics as impaired capillary microcirculation, chronic fatigue syndrome (CFS), proinflammatory cytokines, and functional autoantibodies targeting G-protein-coupled receptors (GPCR-AAbs). Here, we present a case report of successful healing of LCS with BC 007 (Berlin Cures, Berlin, Germany), a DNA aptamer drug with a high affinity to GPCR-AAbs that neutralizes these AAbs. A patient with a documented history of glaucoma, recovered from mild COVID-19, but still suffered from CFS, loss of taste, and impaired capillary microcirculation in the macula and peripapillary region. He was positively tested for various targeting GPCR-AAbs. Within 48 h after a single BC 007 treatment, GPCR-AAbs were functionally inactivated and remained inactive during the observation period of 4 weeks. This observation was accompanied by constant improvement of the fatigue symptoms of the patient, taste, and retinal capillary microcirculation. Therefore, the removal of GPCR-AAb might ameliorate the characteristics of the LCD, such as capillary impairment, loss of taste, and CFS.

## Introduction

Severe acute respiratory syndrome coronavirus 2 (SARS-CoV-2) caused a pandemic with global healthcare issues. The symptoms during acute infection and even after Coronavirus disease 2019 (COVID-19) and long-Covid-syndrome (LCS) are a challenge for each clinician. Even the clinical feature of LCS summarizes a wide range of symptoms; it can be assumed that diversity of molecular mechanisms is the basis for different clinical characteristics of LCS (e.g., pulmonary restriction, endocarditis, malaise, taste loss, and cognitive impairment). One of these clinical entities might be induced by autoimmune mechanisms, being proposed to be involved during infection and thereafter (1). Since not every patient with COVID-19 disease will suffer from LCS afterward, there might be a genetic susceptibility as the basis for the hyperstimulation of the immune system (2). Activation of mast cells, the release of proinflammatory cytokines, the formation of neutrophil extracellular traps (NETosis), and generation of autoantibodies (AAbs) can occur in this complex immune and autoimmune interplay (3–8). Especially, AAbs targeting G-protein coupled receptors (GPCR-AAbs) were observed in the sera of patients recovered from acute COVID-19, with an apparent link to blood microcirculation (8). Recent studies by noninvasive optical coherence tomography angiography (OCT-A) suggested that even months after COVID-19, ocular capillary microcirculation is impaired (9, 36). We propose that the capillary impairment might be induced in part by the agonistic GPCR-AAb targeting ß2-adrenergic receptor (ß2-AR). This link has already been established in clinical studies for patients with glaucoma (37), which serve now as the basis to identify GPCR-AAbs as a potential therapeutic target in patients with impaired capillary microcirculation after COVID-19. Here, we present a case report of a patient with high levels of GPCR-AAbs, impaired retinal capillary microcirculation, and corresponding clinical symptoms of LCS. The patient was monitored before and after the neutralization of the GPCR-AAbs by a DNA aptamer drug with a high affinity to a GPCR-AAb, namely BC 007.

## Results

### Case Report

**Figure 1:**
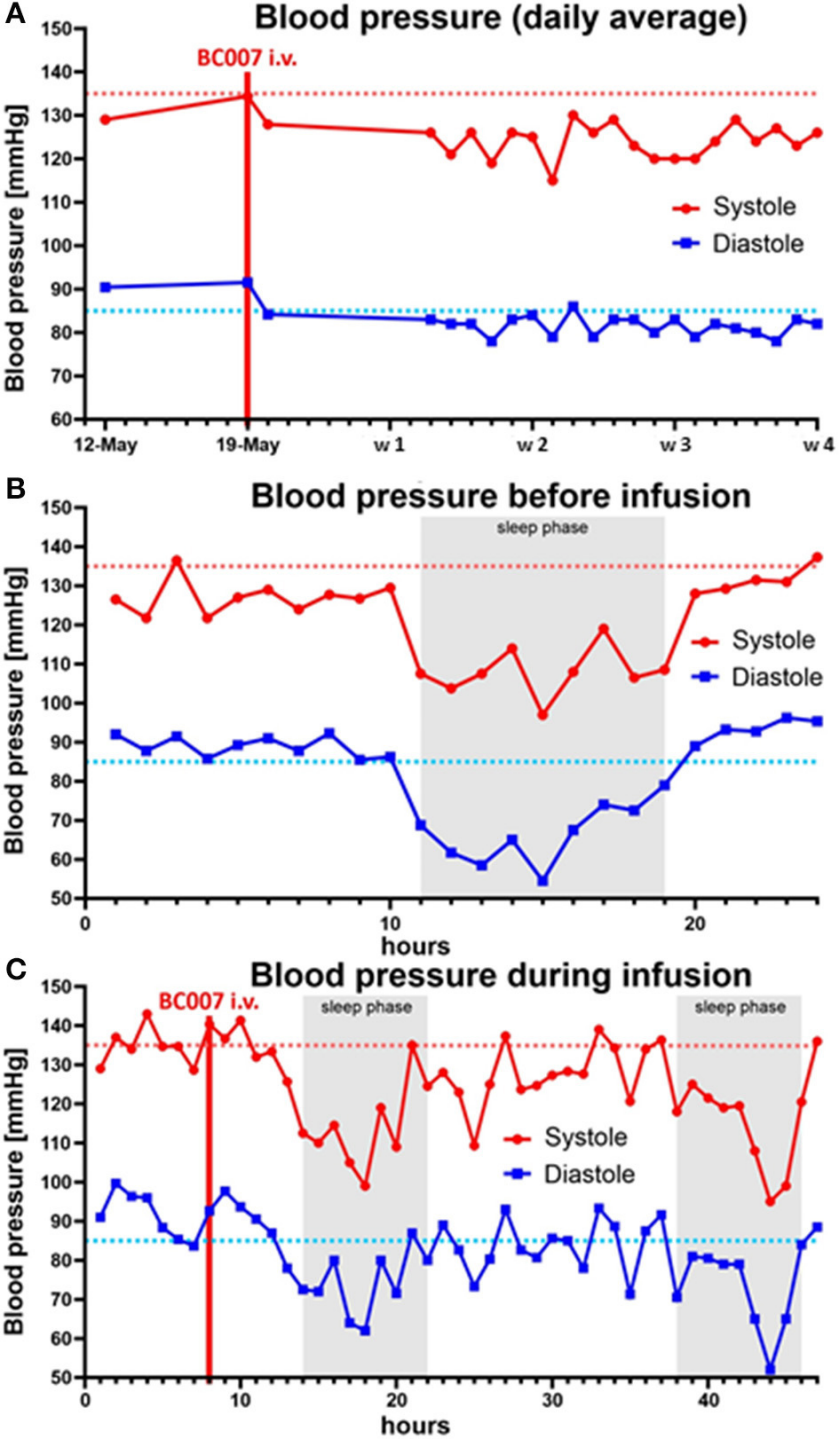
Blood pressure (systolic, diastolic, mmHg) before **(A)**, during **(B)**, and after treatment with BC 007 **(C)**: a reduction of the diastolic blood pressure was observed after the elimination of functional active G-protein-coupled receptor autoantibodies [GPCR-AAb; w-week; dotted line: individual target level of systolic (red) and diastolic (blue) blood pressure].

**Figure 2:**
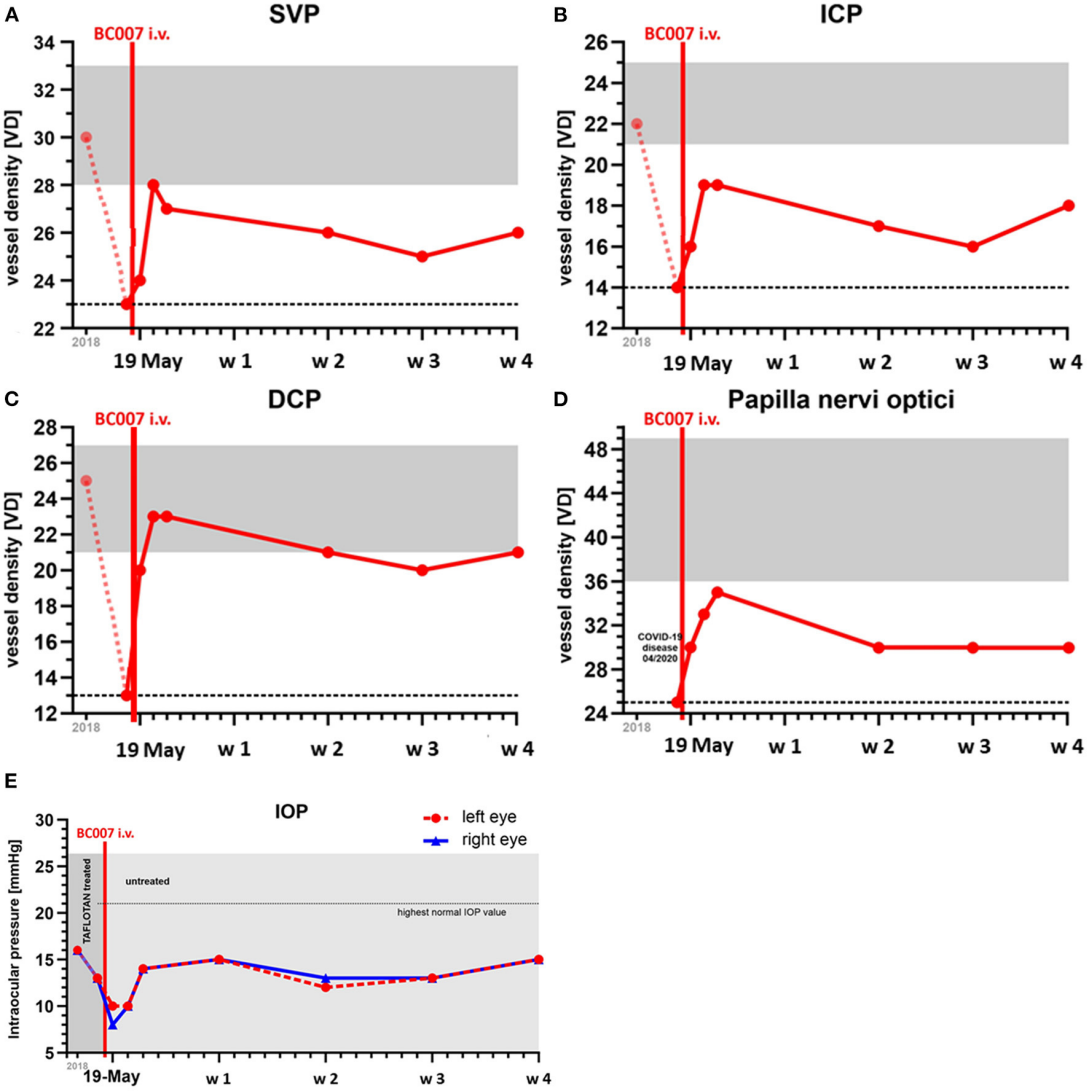
Measurement of ocular parameters (capillary microcirculation, **A–D**; intraocular pressure, **E**): Capillary microcirculation of the macula and peripapillary region, scanned by optical coherence tomography-angiography (OCT-A): vessel density (VD) was monitored in the three layers of the macula (superficial vascular plexus, SVP, **A**; intermediate capillary plexus, ICP, **B**; deep capillary plexus, DCP, **C**) and peripapillary region **(D)**. Vessel density of the macula was reduced after COVID-19 infection compared to the preexisting data of 2018. After treatment with BC 007, the VD increased in all the three layers of the macula and peripapillary region, being stable over at least during the observation period of 4 weeks (gray: range of VD in normal eyes, dotted line: reference level of VD after COVID-19 infection). Data of the left eye are shown as the quality of the OCT-A scans of the right eye were not exploitable due to a hindered fixation of the patient by the presence of cataract. **(E)** Intraocular pressure (IOP, mmHg; measured by Goldmann applanation tonometry) was monitored before and after treatment with BC 007; reference data of 2018 (EGR, ISSN 2191-5008, CS-2011; NTC00494923) were presented: IOP was reduced after treatment with BC 007, as IOP level was stable, yet without IOP lowering eye drops (antiglaucomatous eyes drop before treatment with BC 007: Tafluprost 15 μg/ml, once a day; after treatment with BC 007: without IOP lowering local therapy); w-week; 1st COVID-19 infection: 04/2020; assumed 2nd COVID-19 infection: turn of the year 2021.

**Figure 3:**
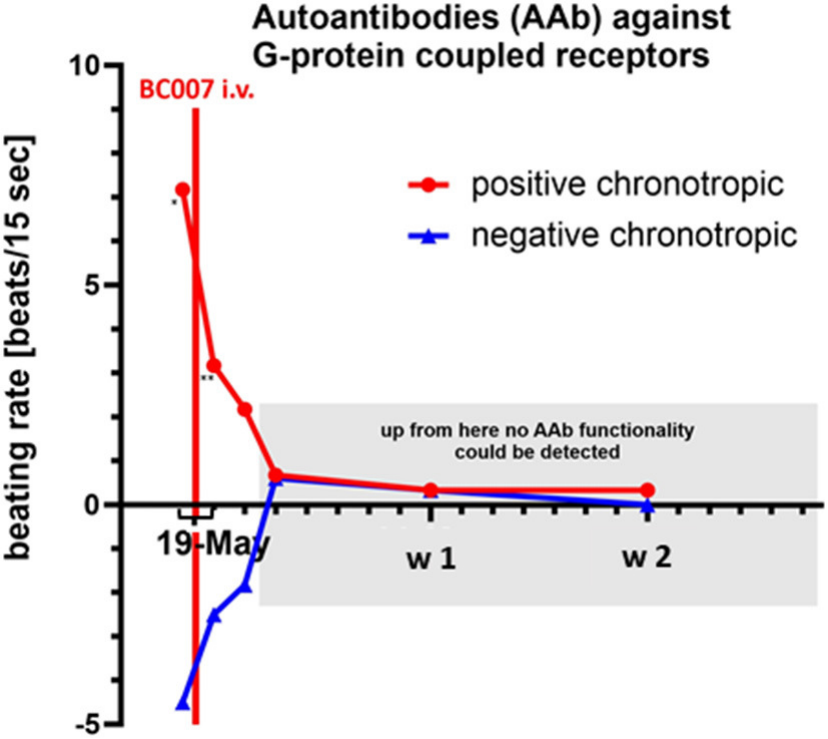
Autoantibodies (AAbs) against G-protein coupled receptors (ß2-AAb, AT-1-AAb, α1-AAb, MAS-AAb, and M2-AAb) before (at the day of infusion, before the treatment, single star) and after treatment with BC 007 [after 2 h (double star), 1 day, 2 days, and 1–4 weeks]: functional activity of the positive and negative chronotrope effect of the different AAbs were reduced even after 2 h and functionally inactive at least over the observation period of 4 weeks (cut-off 1.8 beats/15 s); w-week.

**Figure 4:**
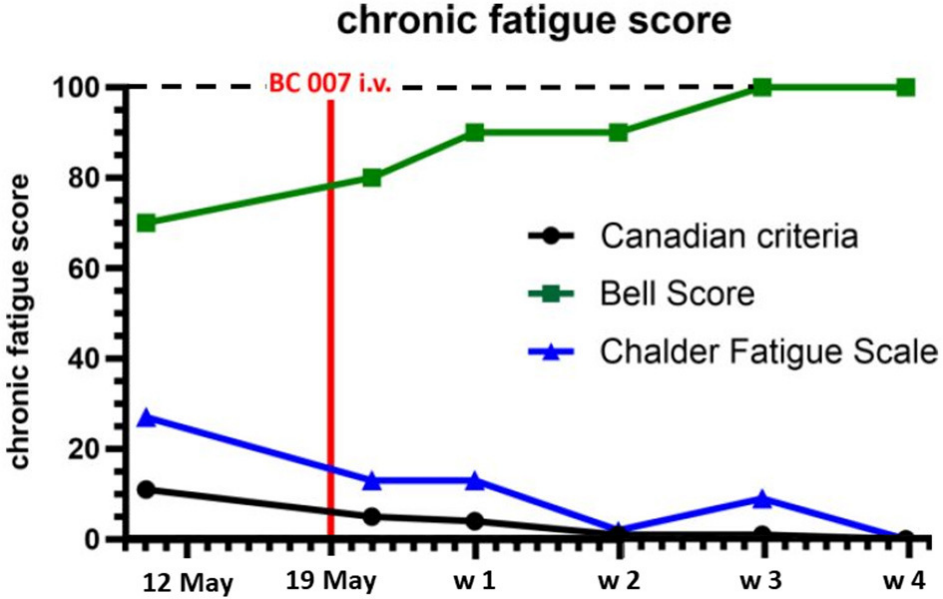
Chronic fatigue syndrome (CFS) was assessed by the Canadian Criteria, Chalder Fatigue Scale, and the Bell Score before and after treatment with BC 007: CFS was reduced after treatment with BC 007, being absent after 4 weeks; the lower is the Canadian Criteria and Chalder Fatigue Scale, the lower is the CFS; the higher the Bell Score, the lower is the CFS; w, week; the range of the scores—Canadian Criteria Score: 0 (best)–34 (worst): Chalder Fatigue Scale: 0 (best)–33 (worst); Bell Score: 100 (best)–0 (worst).

A 59-year old man with a glaucoma history of 17 years suffered from COVID-19 infection two times. No severe symptoms were monitored during both COVID-19 infections regarding the necessity of hospitalization. The first COVID-19 infection occurred on 04/2020. Afterward, no symptoms were recorded by the patient. After a surgical restoration due to sleep apnoe (September 2020), loss of taste occurred, which improved over the next months, yet not reaching normality. It was assumed that the patient suffered from a 2nd COVID-19 infection during the turn of the year with deterioration of the loss of taste and a progressive fatigue syndrome starting on January 2021. Two months later, neurological symptoms arose (brain fog, lack of concentration, and imbalances). In May 2021, it was observed that the Canadian Criteria were 11, Chalder Fatigue Scale was 27, and the Bell Score was 70, limiting the everyday life activities of the patient. In addition, diastolic blood pressure was increased (Figure 1). Being a participant of the Erlanger Glaucoma Registry (EGR, ISSN 2191-5008, CS-2011; NTC00494923) of the Department of Ophthalmology, University of Erlangen-Nürnberg, seropositivity of AAb targeting ß2-AAb has already been known to preexisting for 12 years. After the 1st COVID-19 infection, the preexisting AAb pattern of the patient persisted and additional AAbs targeting Angiontensin-1 receptor (AT-1-AAb), α1-adrenergic receptor (α1-AAb), MAS receptor (MAS-AAb), and muscarinic2-receptor (M2-AAb) were monitored in a cardiomyocyte bioassay. Best-corrected visual acuity (BCVA) was found to be 0.8 (decimal, right eye, RE) and 0.6 (decimal, left eye, LE). Intraocular pressure (IOP) was found to be 13 mm Hg (RE) and 13 mm Hg (LE), respectively, under topical anti-glaucomatous eye drops (Tafluprost 15 μg/ml; once a day). In addition, an Nd:YAG iridotomy, trabeculectomy, and cataract surgery had already been performed on the LE. Optic disc displayed glaucomatous alterations [RE: I, LE: II, classified after Jonas (10, 11)]. The mean defect was found to be 5.6 dB (RE) and 7.0 dB (LE), respectively. Optical coherence tomography–angiography (OCT-A) (Spectralis II OCT-A, Heidelberg Engineering, Heidelberg, Germany) yielded an impaired capillary microcirculation in the macula and peripapillary region compared to normative data (Figures 2A–D). No vaccination against SARS-CoV-2 was done previous a treatment with BC 007. Considering the seropositivity of ß2-AAb, AT-1-AAb, α1-AAb, MAS-AAb, and M2-AAb and their potential link to the impaired retinal microvasculature, together with the patient, we decided to perform an attempt to heal with the aptamer BC 007 (Berlin Cures GmbH, Berlin, Germany). The aim was to (1) eliminate AAbs against GPCR-AAbs, and to (2) improve the impaired capillary microcirculation, measured by OCT-A. In addition, we intended to improve chronic fatigue syndrome (CFS). After and during the infusion of BC 007 (1350 mg in NaCl; for 75 min, supine position), no severe side effects were observed. Seropositivity for ß2-AAb, AT-1-AAb, α1-AAb, MAS-AAb, and M2-AAb was evaluated (Figure 3). Retinal capillary microcirculation was measured by OCT-A as retinal vessel density serves as a correlate for the systemic microcirculation (Figures 2A–D). Chronic fatigue symptoms were assessed by three standardized scores (Canadian Criteria, Chalder Fatigue Scale, and the Bell Score; Figure 4). As early as 2 h after infusion of BC 007, the activity of ß2-AAb, AT-1-AAb, α1-AAb, MAS-AAb, and M2-AAb was reduced and macula and peripapillary vessel density increased. One day after the infusion of BC 007, the GPCR-AAbs were observed to be in the borderline, and after 2 days, no functional activity was observed for all AAbs in the cardiomyocyte bioassay (12). The patient remained seronegative for ß2-AAb, AT-1-AAb, α1-AAb, MAS-AAb, and M2-AAb as long as 4 weeks after the infusion of BC 007 (Figure 3). The improvement of the retinal microcirculation, observed shortly after the infusion of BC 007, was continuously increasing at days 1 and 2 and remained at a constantly increased level in ex ante comparison during the observation period of 4 weeks (Figures 2A–D).

Corresponding to the increased retinal capillary microcirculation, the fatigue symptoms of the patient improved steadily (Figure 4). The brain fog, lack of concentration, and imbalances improved continuously and were absent after 4 weeks. Loss of taste dissolved. As a positive side effect, we observed a lowering of the intraocular pressure (IOP) in both eyes (Figure 2E). The IOP showed a stable follow-up, now without the preexisting topical glaucoma therapy. Actually, during the observation period of 4 weeks, the patient was relieved from anti-glaucomatous medication. In addition to that, previously elevated diastolic blood pressure was lowered (Figure 1).

## Discussion

Corona Virus Disease 2019 infection ranked as a pandemic healthcare issue during 2020. In addition to the clinical features during acute SARS-CoV-2 infection (e.g., pulmonary complications, vasculitis, and thromboembolism), patients often suffer from LCS after recovery. Recent studies discussed the involvement of autoimmunity during and after COVID-19 infection. Microthrombosis (immune thrombosis) and the inflammation itself are triggered in this complex pathophysiology (13–15), potentially mediated by several interacting factors (e.g., IL-1 mediated thromboxane B2 release, IL-6, endothelial cell activation, neutrophil extracellular trap (NET) formations (7, 16–18). Different functional active and inactive AAbs were observed in the sera of patients with COVID-19, targeting 52 and 60 kDa SSA/Ro (19) and interferon-α (20). Functionally active (i.e., agonistic) AAbs are of major interest considering their harmful potential, and they were observed in the sera of patients after COVID-19 (8). Surprisingly, each of these GPCR-AAbs (ß2-AAb, AT-1-AAb, α1-AAb, MAS-AAb, and M2-AAb) target vasoactive receptors. A link of ß2-AAb to the retinal microvasculature was recently observed in patients with glaucoma (37). Patients with agonistic ß2-AAb showed an impaired foveal-avascular zone (FAZ, scanned with OCT-A), focusing on the intermediate capillary plexus. There are only two regions in living humans, at which capillary microvessels can be scanned, monitored, and quantified noninvasively: (1) nailfold by microscopy, (2) retina by OCT-A. OCT-A is a novel technique, enabling noninvasive scans of the retinal capillary system with a high resolution (low μm range). Up to now, there is only one OCT-A device available (Spectralis II OCT-A), scanning the region of interest in three microvascular layers. Those microvascular OCT-A scans correlate well with the human anatomy (21). A previous study showed that especially in the intermediate capillary plexus (ICP) and peripapillary region, a significantly decreased vessel density (VD) was observed after COVID-19 compared to healthy eyes (36). In cases of severe COVID-19 during acute infection, the adjacent retinal microvascular layers were impaired as well (SVP, DCP). In addition, these retinal findings were linked to clinical severity markers (e.g., the highest level of D-dimer and the highest level of Glutamat-Pyruvat-Transaminase). Thus, we postulate that the impaired retinal and peripapillary microcirculation can serve as a correlate for the state of the systemic capillary impairment with possibly different focal expression after SARS-CoV-2 infection. We hypothesize that functional vasoactive GPCR-AAbs contribute to the impairment of the microcirculation. In addition, given the role of ß-adrenergic receptors in stimulating IL-6 production (22), it is plausible that the neutralization of GPCR-AAbs might additionally reduce residual IL-6-mediated inflammation if present.

This molecular pathway is certainly not specific to COVID-19. Patients with glaucoma are known to display agonistic ß2-AAb, being linked to the increased IOP (23, 24) as an unspecific immuneapheresis eliminate the functional active ß2-AAb accompanied by a decrease in IOP and/or a number of antiglaucoma eye drops (23). AT-1-AAb was reported previously in sera of patients with malignant hypertension or preeclampsia (25, 26). ß2-AAb and M2-AAb have already been observed in patients with CFS, independent of COVID-19 (27). Thus, we assume that there are various triggers, stimulating the immune system (e.g., inflammation and ischemia) in order to generate GPCR-AAbs. These coexisting factors could be set up as a vicious circle, as GPCR-AAbs have been observed to act particularly in ischemic or inflammatory regions (28). Binding and neutralizing the GPCR-AAbs would be a great option to eliminate their functional activity in diseases with seropositivity of GPCR-AAbs (23).

BC 007 is an aptamer that binds and neutralizes various GPCR-AAbs. It is an unmodified 15-mer DNA-based oligonucleotide (aptamer), consisting of nine guanosine (purine nucleoside), and six thymidine (pyrimidine nucleoside) nucleosides of the following sequence 5′-GGT TGG TGT GGT TGG-3′. It was identified to neutralize a broad spectrum of pathogenic GPCR-AAbs (29, 30). BC 007 is currently in a phase-2 clinical trial for the treatment of heart failure in patients who are seropositive for ß1-AAb. BC 007 has a favorable safety profile and is well-tolerated as an intravenous infusion of up to 1900 mg i.v. over 75 min, as tested in a dose-finding study. A transient anticoagulatory effect (mild-to-moderate increase in PTT, PT, and INR) that normalizes immediately after the end of infusion was an expected side reaction. The attempt of healing with BC 007 was done as the severity of the LCS increased during 5 months after the 2nd COVID-19 infection (i.e., 13 months after the 1st COVID-19 infection). Thus, we assume that the reduction of LCS is due to the effect of BC 007-mediated neutralization of GPCR-AAbs and not due to an infection resolution. Previous *in vitro* experiments suggested that BC 007 does not bind and neutralize specific anti-SARS CoV-2 antibodies (31). As the patient, presented in this manuscript, was seronegative for specific anti-SARS CoV-2 antibodies previous to the treatment with BC 007, we cannot address this issue.

In conclusion, here, we report a remarkable reversal of an LCS with a variety of characteristics in a patient with seropositivity for GPRC-AAbs by neutralization of these GPCR-AAbs with the aptamer, BC 007. The specific neutralization of the latter improved the impaired retinal capillary microcirculation and chronic fatigue symptoms, arose from LCS. In parallel, loss of taste, imbalances, and brain fog disappeared, and IOP and blood pressure (diastolic) could be lowered.

## Methods

### Cardiomyocyte Bioassay

An established sensitive bioassay was used for the detection of the GPCR-AAbs as ELISA yielded a high specificity in animal samples, yet not in human samples (32). Spontaneously, beating rat cardiomyocytes were cultured for 4 days and then used for the experiments as described in detail (33). The beating rate was counted on six selected fields of the culture flask placed on a heated stage of an inverted microscope (37°C) for 15 s. The AAb containing IgG preparation in the sera of the patients was used in a dilution of 1:50. The cardiomyocyte bioassay was incubated with the sera of the patient (for 60 min; dilution of 1:40). The cut-off was found to be 1.8 beats/15 s.

### Chronic Fatigue Scores

Chronic fatigue (CF) scores were used as follows: Canadian Criteria, Chalder Fatigue Scale, and the Bell Score. The more are the decrease of the Canadian Criteria and Chalder Fatigue Scale, the merrier is the CF symptoms of the patient. The more is the increase of the Bell Score, the merrier is the CFS symptoms.

### Optical Coherence Tomography–Angiography

Retinal vessels density (VD) was scanned with the Heidelberg Spectralis II (Heidelberg Engineering, Heidelberg, Germany). Two anatomical areas of the eye were monitored (scan angle: 15° × 15°; scan size: 2.9 × 2.9 mm; total scan size: 8.41 mm^2^; diameter of inner ring: 0.8 mm; diameter of the outer ring: 2.9 mm): macula and peripapillary region. By scanning the macula region, a subdivision of the region into three microvascular layers can be done: superficial vascular plexus (SVP, thickness: 80 μm), intermediate capillary plexus (ICP, thickness: 50 μm), and deep capillary plexus (DCP, thickness: 40 μm). Peripapillary microvessels were scanned in one overall layer as provided by the company. The scans were done in the highest commercially available lateral resolution of 5.7 μm/pixel. After manual checking for correct segmentation or artifacts, analysis of the scans was done by the Erlangen-Angio Tool (EA-Tool; version 3.0; Matlab, The MathWorks, Inc., R2017b), enabling quantification of VD with high reproducibility and reliability (34). The EA-Tool (version 3.0) is advanced software, implementing an Anatomical Positioning System (APS, part of Glaucoma Module Premium Edition [GMPE], Heidelberg Engineering, Germany). The APS module enables an exact alignment of OCT-A scans to individual Fovea-to-Bruch's Membrane Opening-Center (FoBMOC) axis of each patient (“APS-ify”). In addition, the advanced EA-Tool (version 3.0) implements the option to analyze peripapillary VD according to the BMO landmarks (BMO-based peripapillary VD), representing the exact border of the optic nerve head. The BMO and APS coordinates were exported by SP-X1902 software (prototype software, Heidelberg Engineering, Heidelberg, Germany) (35).

## Data Availability Statement

The raw data supporting the conclusions of this article will be made available by the authors, without undue reservation.

## Ethics Statement

Ethical review and approval was not required for the study on human participants in accordance with the local legislation and institutional requirements. The patients/participants provided their written informed consent to participate in this study. Written informed consent was obtained from the individual(s) for the publication of any potentially identifiable images or data included in this article.

## Author Contributions

All authors listed have made a substantial, direct and intellectual contribution to the work, and approved it for publication.

## Conflict of Interest

BH and CM: Heidelberg Engineering. AH, PG, JM, and GW employed by Berlin Cures GmbH, shareholders of Berlin Cures Holding AG, the holding company of Berlin Cures. The remaining authors declare that the research was conducted in the absence of any commercial or financial relationships that could be construed as a potential conflict of interest.

## Publisher's Note

All claims expressed in this article are solely those of the authors and do not necessarily represent those of their affiliated organizations, or those of the publisher, the editors and the reviewers. Any product that may be evaluated in this article, or claim that may be made by its manufacturer, is not guaranteed or endorsed by the publisher.
